# Differential Expression of Alpha 4 Integrins on Effector Memory T Helper Cells during *Bordetella* Infections. Delayed Responses in *Bordetella pertussis*

**DOI:** 10.1371/journal.pone.0052903

**Published:** 2012-12-27

**Authors:** Tuan M. Nguyen, Dipti Ravindra, Brian Kwong, Sana Waheed, Ryan Ferguson, Nicole Tarlton, Victoria Wu, Christopher S. Sequeira, Martina Bremer, Tzvia Abramson

**Affiliations:** 1 Department of Biology, San Jose State University, San Jose, California, United States of America; 2 Department of Mathematics, San Jose State University, San Jose, California, United States of America; Universidad Nacional de La Plata, Argentina

## Abstract

*Bordetella pertussis* (*B. pertussis*) is the causative agent of whooping cough, a respiratory disease that is reemerging worldwide. Mechanisms of selective lymphocyte trafficking to the airways are likely to be critical in the immune response to this pathogen. We compared murine infection by *B. pertussis*, *B. parapertussis*, and a pertussis toxin-deleted *B. pertussis* mutant (*BpΔPTX*) to test the hypothesis that effector memory T-helper cells (emTh) display an altered pattern of trafficking receptor expression in *B. pertussis* infection due to a defect in imprinting. Increased cell recruitment to the lungs at 5 days post infection (p.i.) with *B. parapertussis*, and to a lesser extent with *BpΔPTX*, coincided with an increased frequency of circulating emTh cells expressing the mucosal-associated trafficking receptors α4β7 and α4β1 while a reduced population of these cells was observed in *B. pertussis* infection. These cells were highly evident in the blood and lungs in *B. pertussis* infection only at 25 days p.i. when *B. parapertussis* and *BpΔPTX* infections were resolved. Although at 5 days p.i., an equally high percentage of lung dendritic cells (DCs) from all infections expressed maturation markers, this expression persisted only in *B. pertussis* infection at 25 days p.i. Furthermore, at 5 days p.i with *B. pertussis*, lung DCs migration to draining lymph nodes may be compromised as evidenced by decreased frequency of CCR7^+^ DCs, inhibited CCR7-mediated *in vitro* migration, and fewer DCs in lung draining lymph nodes. Lastly, a reduced frequency of allogeneic CD4^+^ cells expressing α4β1 was detected following co-culture with lung DCs from *B. pertussis*-infected mice, suggesting a defect in DC imprinting in comparison to the other infection groups. The findings in this study suggest that *B. pertussis* may interfere with imprinting of lung-associated trafficking receptors on T lymphocytes leading to extended survival in the host and a prolonged course of disease.

## Introduction

Despite widespread vaccination against *Bordetella pertussis* (*B. pertussis*), whooping cough is reemerging with high rates of morbidity and mortality worldwide [1]. In the U.S., several states have declared pertussis epidemics. Due to waning efficacy of the acellular vaccine, immunized adults may acquire whooping cough and manifest only a mild disease that may pass undetected. However, they serve as reservoirs of infection for unimmunized infants who are at risk of developing a profound and deadly respiratory disease. Thus, whooping cough continues to be a worldwide problem, and a better understanding of *B. pertussis* pathogenesis would lead to improved treatment and prevention.

*Bordetella pertussis* is a highly contagious human pathogen that colonizes the upper respiratory tract by adhering to ciliated bronchial epithelial cells. In addition to the characteristic “whoop”-like cough in infants, hallmarks of disease are extreme lymphocytosis and a prolonged course of disease with a recovery period of up to several months. A variety of potent virulence factors work in concert to contribute to the unique pathogenesis of *B. pertussis* [2]. Specifically, pertussis toxin (PTX) is linked to the extreme lymphocytosis and to the pathology associated with delayed recovery from the disease [3], [4].

Pertussis toxin, a secreted soluble toxin, is composed of several subunits [5]. The catalytic A subunit (S1) is well documented to inhibit G-protein coupled receptors (GPCR) [6] and chemo-attractant responses in host cells [7]. The B subunit (PTX-B) plays a more diverse role; it is documented to facilitate *B. pertussis* colonization and the binding of soluble PTX to a large variety of glycosylated cellular receptors [8]. Previous studies have shown that PTX acts early post infection (p.i.) to promote efficient bacterial colonization of the airways [9],[10] and to delay neutrophil recruitment to the lungs. This delay is primarily associated with the early effects of PTX on chemokine expression in the lung [11], and reduced extravasation of neutrophils through pulmonary endothelial cells [12].

Despite ample documentation of the innate immune response during infection with *B. pertussis*, less information is available on the adaptive immune response in this disease. *In vitro* studies demonstrate that PTX-B is responsible for the desensitization of the chemokine receptor CXCR4 in Jurkat cells [13]. More recently, PTX-B was shown to have lectin-like activity, causing the activation of T cell receptors (TCRs) through cross-linking [14]. Wild type *B. pertussis* was shown to induce a late Th1/Th17 response that eventually leads to the resolution of *B. pertussis* infection [15]. However, much less studied are the chemo-attractant receptors [generally termed “trafficking receptors” (TRs)] and homing capabilities of T helper cells to the airways and lungs in this infection.

Specific combinations of TRs that direct lymphocytes to the gut and skin in a selective fashion are well documented [16], [17], [18]. Currently, no specific TR pattern has been identified for exclusive trafficking to the respiratory system [19]. However, it has been reported that lymphocytes recirculating through the lungs express lower levels of α4β7 compared to intestinal lymphocytes [18] and high levels of α4β1 (VLA-4) [20], collagen-binding very late antigen-1 (VLA-1 or α1β1) [21], leukocyte function-associated antigen-1 (LFA-1 or CD11a) [22], CD49f (α6) and P-selectin ligand (Psel-lig) [23], [17], [18], [24], [25].

In this study, we hypothesized that despite the extreme lymphocytosis observed during infection with *B. pertussis*, circulating lymphocytes display an altered pattern of TR expression due to an imprinting defect, leading to a delay in migration to the lung and a prolonged state of disease.

We tested this hypothesis using a murine model of *Bordetella* which partially mimics human pathology and is widely used for the study of pathogenesis and vaccine development [26]. We compared two natural strains that normally infect humans: *B. pertussis* and *B. parapertussis*. Unlike *B. pertussis*, *B. parapertussis* triggers a milder and shorter respiratory disease in humans [27]. While these two species evolve from common progenitors and share 90% genome homology [28], [29], *B. parapertussis* genome lacks the expression of several important virulence factors including PTX [30], [31]. Although a PTX-deleted *B. pertussis* strain (*BpΔPTX*) is available for evaluating PTX function, this engineered strain manifests reduced colonization compared to *B. pertussis* [9]. Unlike *BpΔPTX*, *B. parapertussis* utilizes other bacterial factors to efficiently colonize the host [32]. Thus, we primarily tested the hypothesis of this study using the two natural pathogens and confirmed PTX-dependent effects using *BpΔPTX*.

The findings of this study suggest that α4 integrins, in combination with β7 and β1, play an important role in emTh cell migration during *B. pertussis* infection. Altered imprinting of these TRs may be targeted by *B. pertussis* in order to promote survival in the host and a prolonged state of disease.

## Materials and Methods

### Animals

All studies using mice were approved by the Institutional Animal Care and Use Committee (IACUC) at San José State University, San José, CA (protocol # 921), and all animals were handled in accordance with institutional guidelines. Female BALB/c (Thy1.2^+^) mice and female FVB/N (Thy1.1^+^) mice were purchased from Simonsen Labs (Gilroy, CA).

### Bacteria

The following *Bordetella* strains were used: *B. pertussis* 338, which is a nalidixic acid-resistant derivative of Tohama I [33]; *B. parapertussis* (ATCC 9305); and *BpΔPTX*, which is BPTOX6 (ptx*Δ*6, a derivative of parental strain BP338) and has a deletion of the pertussis toxin operon and a substitution of a kanamycin-resistance cassette at that location [34]. *Bordetella* strains were grown on Bordet-Gengou blood agar plates, and single colonies were used to inoculate Stainer-Scholte broth supplemented with (2, 6-di-O-methyl) beta-cyclodextrin (Heptakis) (Sigma-Aldrich, St. Louis, MO), casamino acid, and proline two days prior to experimental infection as previously described [35].

### Infection

**Figure 1:**
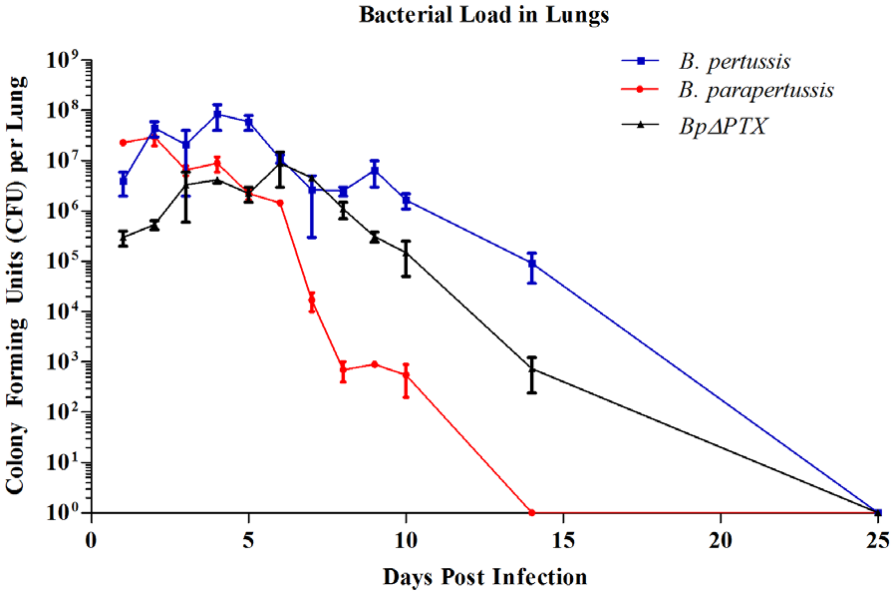
*B. pertussis* induces a longer course of infection than *B. parapertussis*. BALB/c mice were infected intranasally with 5×10^6^ CFU of *Bordetella* strains in 20 µl of PBS, or mock infected (as detailed in the Methods section). Bacterial load in the lungs was assessed at the time points shown above. 3 mice were used at each time point. This graph represents data from two independent experiments.

Six-week-old female BALB/c mice were infected intranasally (i.n.) with 5×10^6^ colony forming units (CFU) of either *B. pertussis*, *B. parapertussis*, or *BpΔPTX* in 20 µl of phosphate buffered saline (PBS). Uninfected control mice received 20 µl PBS [36]. Two time points of experimental infection were established: 5 days p.i. (early stage) and 25 days p.i. (late stage). These time points were previously determined through a time-course experiment in which bacterial load in the lungs was measured consecutively for 10 days, and again on 14 days and 25 days p.i. (Fig. 1). In brief, lungs from experimentally infected mice were harvested, homogenized, and plated on Bordet-Gengou plates in order to confirm colonization of the lungs and assess the bacterial load.

### Preparation of blood leukocytes: dextran sedimentation

Leukocyte suspensions were prepared by dextran sedimentation as previously described [37]. In brief, whole blood from infected or control mice was obtained by cardiac puncture and mixed with a solution containing 1.5 mM EDTA and 2% dextran, and incubated at 37°C for 30 minutes. Leukocyte-rich plasma, located on the top of the aggregated erythrocytes, was carefully collected and centrifuged at 300×g for 5 minutes. The cell pellets were mixed with 3 ml red blood cell lysis buffer (Sigma-Aldrich, St. Louis, MO) for 5 minutes and washed with RPMI-1640 containing 10% fetal calf serum (FCS). Cells were stained for flow cytometry analysis or used for *in vitro* migration and adhesion assays.

### Preparation of lung mononuclear cells

Lungs from infected or control BALB/c mice were perfused with 30–50 ml of PBS. The lungs and trachea were removed, processed, and digested in RPMI-1640 media containing 10% FCS, 75 U/ml DNase I (Sigma-Aldrich) and 250 U/ml Type I collagenase (Worthington Biochemical Corporation, Lakewood, NJ) in a 37°C orbital shaker for 45–60 minutes (5 ml media/lung). The digested lungs were passed through a 70 µm nylon mesh strainer, and cells were washed with RPMI-1640 to remove residual digest medium. Digested lung tissue was resuspended in 40% Percoll (GE Lifescience, Piscataway, NJ), slowly underlaid with 70% Percoll, and centrifuged at 300×g for 25 minutes (without brakes) to create a density gradient. Cells at the 40–70% interface (mononuclear cells) were collected and stained for flow cytometry analysis, or further used for DC enrichment.

### Enrichment of lung DCs

Lung mononuclear cells from infected or control mice, obtained via Percoll density gradient centrifugation, were enriched for DCs (CD11c^+^ cells) by positive selection using directly conjugated anti-CD11c magnetic microbeads (N418) with a MiniMACS separator (Miltenyi Biotech, Auburn, CA). In brief, lung mononuclear cells were incubated with 100 µl CD11c microbeads for 15 minutes at 4°C. Cells were then washed with MACS buffer (PBS, 0.5% FCS, 2 mM EDTA) and resuspended in 500 µl of the same buffer. Cells were loaded on one MACS MS column, which enriched for CD11c^+^ cells. The cells were either stained for flow cytometry analysis or used in co-culture experiments.

### Splenocyte preparation

The spleens of 6-week-old female FVB/N Thy1.1^+^ mice were harvested and pressed through a nylon mesh to prepare a single cell suspension [38]. Splenocytes were incubated with 3 ml red blood cell lysis buffer (Sigma-Aldrich) for 5 minutes and washed with RPMI-1640 containing 10% FCS. Cells were centrifuged and resuspended in PBS containing 0.5% bovine serum albumin (BSA).

### Enrichment of allogeneic CD4^+^ T cells

Splenocytes obtained from female FVB/N Thy1.1^+^ mice were enriched for CD4^+^ T cells by positive selection using directly conjugated anti-CD4 microbeads (L3T4) with a MidiMACS or QuadroMACS separator (Miltenyi Biotech). In brief, splenocytes were incubated with 200 µl of CD4 microbeads for 15 minutes at 4°C. Cells were then washed with MACS buffer (PBS, 0.5% FCS, 2 mM EDTA) and resuspended in 500 µl of the same buffer. Cells were loaded on one MACS LS column, which was used to enrich for CD4^+^ cells.

### Co-culture experiments

Lung DCs enriched by CD11c^+^ positive selection were co-cultured in a 24-well flat-bottomed plate with positively selected CD4^+^ cells from naïve FVB/N Thy1.1^+^ allogeneic mice (1∶5 ratio), in 500 µl RPMI-1640 media with 10% FCS and 1% of penicillin and streptomycin (RPMI complete). Cells were co-cultured for 4 days at 37°C with 5% CO_2_ to allow for T cell proliferation and trafficking receptor imprinting, and were subsequently analyzed via flow cytometry.

### T cell proliferation experiments

T cell proliferation was determined by Carboxyfluorescein Diacetate Succinimidyl Ester (CFSE) (Invitrogen, Grand Island, NY) dye dilution. Briefly, positively selected CD4^+^ cells from allogeneic FVB/N Thy1.1^+^ mice were labeled with CFSE as previously described [39]. Labeled cells were co-cultured for 4 days with positively selected lung DCs (CD11c^+^) in 500 µl RPMI complete media at 37°C with 5% CO_2_, followed by flow cytometry analysis.

### Flow cytometry

Lung-derived DCs were stained with antibodies as indicated in Table 1, and T cells were stained with antibodies as indicated in Table 2 (BioLegend, San Diego, CA; eBioscience, San Diego, CA; BD Biosciences, San José, CA). Stained cells were acquired on an LSRII flow cytometer (BD Biosciences) at the Stanford University FACS facility (Stanford, CA), and analyzed using FlowJo analysis software (Tree Star Inc., Ashland, OR). The frequencies of cells expressing the above trafficking markers were compared for *B. pertussis*, *B. parapertussis*, and *BpΔPTX* infections and were normalized to uninfected controls as detailed in the figure legends.

**Table 1:** Antibodies used to stain dendritic cells.

Marker	Clone	Source
CD11c	N418	BioLegend
CD11b	M1/70	BioLegend
MHC-II	M5/114.15.2	BioLegend
CD86	GL-1	BioLegend
CCR7	4B12	BioLegend
CXCR4	2B11	eBiosicence

**Table 2:** Antibodies used to stain T cells.

Memory/Activated T cell Markers			Trafficking Receptors		
Marker	Clone	Source	Marker	Clone	Source
CD4	RM4-5	BD Biosciences	CD29 (β1)	HMb1-1	BioLegend
CD38	90	BioLegend	CD49d (α4)	9C10	BioLegend
Thy1.1	OX-7	eBioscience	β7	FIB27	BioLegend
CD44	IM7	BioLegend	CD11a	M17/4	BioLegend
CD45RB	C363-16A	BioLegend	CD162	2PH1	BD Biosciences

### Hematoxylin and eosin staining for histology of lung sections

The lungs of control and infected BALB/c mice were infused with PBS and Optimal Cutting Temperature (OCT) compound embedding media (1∶1 ratio) and then frozen in OCT media on dry ice. The lungs were cut into 6 µm sections and fixed with cold acetone. They were subsequently stained with hematoxylin and eosin (H&E). Ten randomly selected fields (400×) from each lung were analyzed.

### Immunofluorescence of lung sections

*Macrophage and B cell staining*: Acetone-fixed frozen lung sections were washed with 1X tris-buffered saline (TBS) containing 0.1% Tween 20, and blocked with PBS containing 5% goat serum (Santa Cruz Biotechnology, Santa Cruz, CA) for 30 minutes. The sections were sequentially incubated with F4/80 rat anti-mouse monoclonal antibody (mAb) (CI:A3-1, 1∶200 dilution for 1 hour) or rat IgG2b isotype (RTK4530, 1∶200 dilution for 1 hour), FITC goat anti-rat antibody (Jackson ImmunoResearch, Westgrove, PA, 1∶500 dilution for 30 minutes), PBS with 5% rat serum (Lampire Laboratory, Pipersville, PA for 30 minutes), goat anti-rat IgG Fab fragment (Jackson ImmunoResearch, 1∶200 dilution for 30 minutes), CD45R/B220 rat anti-mouse mAb (RA3-6B2, 1∶200 dilution for 1 hour) or rat IgG2a isotype (RTK2758, 1∶200 dilution for 1 hour), and Dylight 594 goat anti-rat antibody (Jackson ImmunoResearch, 1∶500 dilution for 30 minutes). Slides were counterstained with 4′,6-diamidino-2-phenylindole (DAPI).

*Neutrophil and T cell staining*: Acetone-fixed frozen lung sections were washed with 1X TBS containing 0.1% Tween 20, and blocked with PBS containing 5% goat serum for 30 minutes. Sections were sequentially incubated with CD3 hamster anti-mouse mAb (145-2C11, 1∶200 dilution for 1 hour) or Armenian Hamster IgG isotype (HTK888, 1∶200 dilution for 1 hour), Dylight 488 goat anti-hamster antibody (Poly4055, 1∶500 dilution for 30 minutes), Gr-1 rat anti-mouse mAb (RB6-8C5, 1∶200 dilution for 1 hour) or rat IgG2b isotype (RTK4530, 1∶200 dilution for 1 hour), and Dylight 594 goat anti-rat antibody (Jackson ImmunoResearch, 1∶500 dilution for 30 minutes). Slides were counterstained with DAPI.

Sections were visualized at 100× magnification using a LEICA confocal microscope (DMI-4000B). All antibodies used for immunofluorescence staining were purchased from BioLegend unless otherwise indicated.

### Competitive α4-dependent chemotaxis *in vitro* migration

Chemotaxis assays were performed with transwell tissue culture plates (3 µm pores) purchased from Corning Costar (Tewsbury, MA) as previously described [40]. The transwells were coated with mouse VCAM-1 (100 µg/ml; R&D Systems, Minneapolis, MN) overnight at 4°C and then blocked with 2% BSA for 30 minutes at room temperature. A mixture of blood leukocytes from *B. pertussis*- (CFSE labeled; Invitrogen) [39] and *B. parapertussis*-infected mice (eFluor 670 labeled; eBioscience) [41] at a 1∶1 ratio (0.5×10^5^ cells each) were resuspended in 100 µl of chemotaxis buffer (RPMI-1640, 0.5% BSA) and loaded onto the upper chamber of the transwell. Stromal cell-derived factor-1α (SDF-1α; 100 ng/ml; Miltenyi Biotech) was added to the lower chamber. Neutralizing anti–α4 monoclonal antibody (PS/2, 5 µg/ml) (gift from Dr. Butcher, Stanford) was added to block the cells in selected upper chambers. The cells were allowed to migrate for 4 hours at 37°C, and the cells that migrated to the lower chamber were enumerated by flow cytometry (BD FACS Calibur, BD Biosciences). Of note, the use of a 3 µm pore transwell membrane selects for lymphocytes from blood.

### Cell adhesion assay

A 96-well plate was coated with mouse VCAM-1 (10 µg/ml) overnight at 4°C, and then blocked with 2% BSA for 30 minutes at room temperature. An aliquot of 0.5×10^5^ blood leukocytes from *B. pertussis*- or *B. parapertussis*-infected mice was loaded into the wells. After 40 minutes of incubation at room temperature, the wells were washed 3 times. The adhering cells were stained with 0.2% crystal violet in 10% ethanol. The stain was solubilized using a 50/50 mixture of 0.1 M NaH_2_PO_4_ (pH 4.5) and 50% ethanol, and absorbance was measured at 595 nm on a microplate reader.

### Non-competitive CCR7-mediated chemotaxis assay of CD11c^+^ lung cells

The chemotaxis assay was performed as previously described [42]. In brief, 5×10^5^ CD11c^+^ lung cells in a total volume of 100 µl chemotaxis medium (RPMI-1640, 0.5% BSA) was added to the upper chambers of 24-well transwell plates with 5-µm-pore-size polycarbonate inserts (Corning Costar). 600 µl of chemotaxis medium containing either CCL19 (100 ng/ml, Shenandoah Biotechnology, Warwick, PA), CCL21 (100 ng/ml, Peprotech, Rocky Hill, NJ) or CCL28 (100 ng/ml, Peprotech) were added to the lower chambers. After 5 hours of incubation at 37°C, migrated cells from the lower chambers were collected, stained with anti-CD11c (Biolegend) and anti-MHC-II (Biolegend), and enumerated with Trucount beads (BD Biosciences) by flow cytometry (BD FACS Calibur, BD Biosciences). Results are reported as the chemotaxis index, which is the ratio of transmigrated cells in the presence of ligands divided by the number of transmigrated cells in the absence of ligands.

### Enumeration of CD11c+ cells in the mediastinal lymph nodes

The mediastinal lymph nodes were harvested, pooled for each mouse, and pressed through a nylon mesh to create a single cell suspension. Cells were washed with RPMI-1640, stained with anti-CD45 (30-F11, BD Biosciences) and anti-CD11c (Biolegend), and enumerated with Trucount beads (BD Biosciences) by flow cytometry (BD FACS Calibur, BD Biosciences). In each experiment, the absolute number of CD11c^+^ cells in the mediastinal lymph nodes from individual mice was calculated, and three mice were used per infection. Then, the average number of CD11c^+^ cells for three mice per infection was normalized to that from *B. pertussis* infection within the same experiment (1.0 by definition). Depicted results are combined fold-values from three separate experiments.

### Statistical analysis

Data are presented as mean values ± standard error of the mean (SEM). Statistical significance between sets of data was assessed with the two-tailed Student's *t*-test.

## Results

The experiments of this study were designed to test if infections with *B. pertussis*, *B. parapertussis*, and the mutant strain *BpΔPTX* in mice differ in lymphocyte trafficking characteristics.

### Bacterial load in the lungs of mice infected with *B. pertussis, B. parapertussis*, or *BpΔPTX*

To assess colonization of the lungs and establish a time-course of infection, 6-week-old BALB/c mice were infected by intranasal inoculation with 5×10^6^ CFUs of *B. pertussis*, *B. parapertussis*, or *BpΔPTX*. CFUs in the lung were examined at the various time points indicated in Fig. 1.

Comparably high numbers of CFUs were retrieved up to 5 days p.i. from the lungs of mice infected with *B. pertussis, B. parapertussis* and *BpΔPTX*. However, bacterial load dropped gradually after 5 days p.i. with *B. parapertussis*, and no bacteria was cultured from the lungs by 14 days p.i. At the same time, considerable CFUs were retrieved up to and at 14 days p.i. from *B. pertussis* and *BpΔPTX* infected lungs. No *B. pertussis* or *BpΔPTX* CFU was cultured at 25 days p.i. (Fig. 1).

### Delayed recruitment of leukocytes to the lungs in *B. pertussis* infection

**Figure 2:**
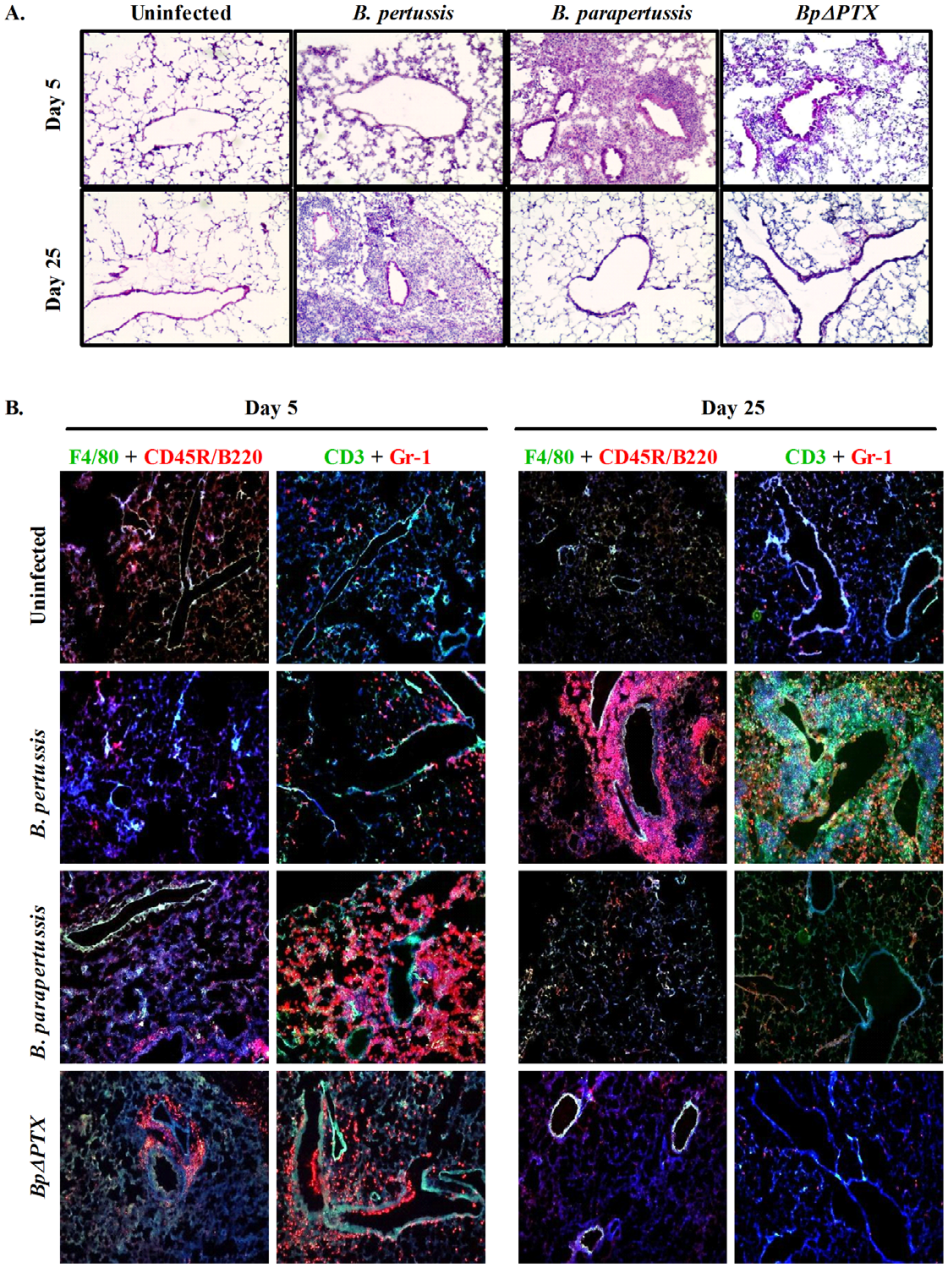
Leukocyte recruitment to the lungs of mice infected with *Bordetella* strains. (**A**) Frozen sections of lungs from BALB/c mice infected with *B. pertussis*, *B. parapertussis*, *BpΔPTX*, or uninfected controls at 5 and 25 days p.i. were H&E stained, and ten independent fields per infection were photographed under 400× magnification. Three independent experiments were performed. (**B**) Frozen lung sections from mice infected with *B. pertussis*, *B. parapertussis*, *BpΔPTX*, or uninfected controls at 5 and 25 days p.i. were stained sequentially with F4/80 (green) for macrophages, followed by CD45R/B220 (red) for B cells. Alternatively, the slides were sequentially incubated with CD3 (green) for T cells, followed by Gr-1 (red) for neutrophils. Slides were counterstained with DAPI for cell nuclei. Staining with isotype control mAbs revealed no false positive cells (not depicted). Ten separate fields were photographed from each treatment at 100× magnification on a confocal microscope.

H&E analyses of lung sections derived from 5 days p.i. with *B. pertussis* showed no significant cell accumulation, similar to corresponding sections from uninfected mice (Fig. 2A). However, at the same time point, the lungs of *B. parapertussis* (and to a lesser degree with *BpΔPTX)* infected mice displayed dense cell infiltration. Conversely, at 25 days p.i., a massive recruitment of cells was apparent in the lungs of mice infected with *B. pertussis* while no cell accumulation was demonstrated in *B. parapertussis or BpΔPTX* infection.

Similarly, immunofluorescent staining of lung sections at 5 days p.i. confirmed that no significant recruitment of cells was detected in *B. pertussis* infection while a predominant infiltration of neutrophils was present in *B. parapertussis* (and to a lesser extent in *BpΔPTX*) infected lungs (Fig. 2B). Again, at 25 days p.i., leukocyte accumulation was confirmed in lung sections from *B. pertussis* infection, where neutrophils occupied the lung parenchyma, and T and B cells clustered mainly around the airways. However, at this time point, no significant accumulation of immune cells was observed in lung sections derived from *B. parapertussis* and *BpΔPTX* infection.

### Expression of mucosal integrin receptors on emTh cells in *Bordetella* infections

**Figure 3:**
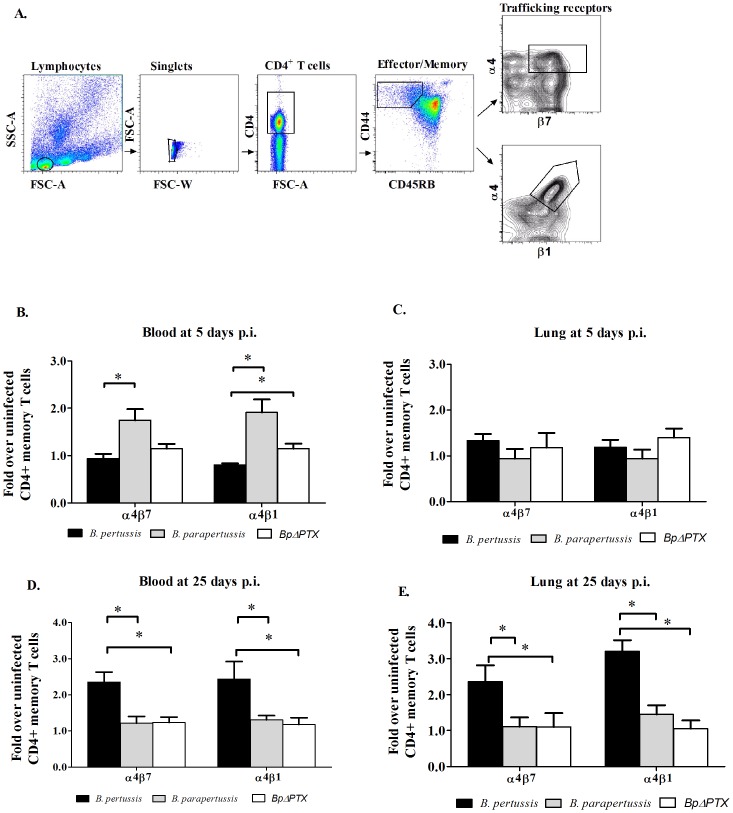
Trafficking receptor expression on emTh cells in blood and lungs from *B. pertussis*-, *B. parapertussis*-, and *BpΔPTX*- infected mice at 5 and 25 days p.i. (**A**) Mononuclear cells were obtained from the peripheral blood and from perfused lungs. Cells were gated for CD4^+^, and further gated for emTh cells (CD44^+^/CD45RB^low^). The frequency of emTh cells expressing trafficking receptors was assessed in the blood (**B, D**) and lungs (**C, E**), and normalized to the respective cells in uninfected control mice (1.0 by definition). The bar charts represent compiled data with error bars showing the SEM of four independent experiments. In each independent experiment, six mice were used per treatment group. *: p<0.05, no asterisk indicates p<0.05 as analyzed by a two-tailed student's *t*-test.

Given the delay in leukocyte recruitment to the lungs early in infection with *B. pertussis* (Fig. 2), we hypothesized that the differential timing of lymphocyte recruitment may be attributed to the altered expression of mucosal associated TRs on circulating and lung resident emTh cells, as defined by CD4^+^/CD44^+^/CD45RB^low^ cells expressing α4β7 and α4β1 (Fig. 3A).

#### Day 5 p.i., blood

The percentage of circulating emTh cells derived from *B. pertussis* infection expressing α4β7 and α4β1 did not differ significantly from corresponding cells of uninfected mice (0.96- and 0.76-fold, respectively) (Fig. 3B). At the same time, in *B. parapertussis* infection, an increase in the percentage of circulating emTh cells that expressed α4β7 and α4β1 was observed, which on average was 1.8- and 1.9-fold more than respective cells from uninfected mice (p<0.05). Although a modest increase in the percentage of emTh cells expressing α4β1 was noted in *BpΔPTX* compared to respective cells derived from *B. pertussis* infection, this percentage did not vary with respect to uninfected mice. No significant difference was detected in the frequency of circulating emTh cells out of CD4^+^ cells between all experimental models (data not shown).

#### Day 5 p.i., lungs

In all models of infection, the proportion of lung emTh cells expressing α4β7 and α4β1 did not differ significantly at this time compared to uninfected cells (Fig. 3C).

#### Day 25 p.i., blood

At this late time point, the pattern of expression was reversed, and an elevated proportion of circulating emTh cells derived from *B. pertussis* infection expressed α4β7 and α4β1 (2.35- and 2.43-fold respectively, p<0.05 compared to uninfected mice). In comparison, the frequency of cells expressing α4β7 and α4β1 from *B. parapertussis* and *BpΔPTX* infection did not differ from uninfected mice (Fig. 3D).

#### Day 25 p.i., lung

Similarly, the frequency of lung emTh cells from *B. pertussis* infection expressing α4β7 and α4β1 increased to 2.36- and 3.21-fold respectively (p<0.05), in contrast to cells from *B. parapertussis* and *BpΔPTX* that did not differ significantly from uninfected mice (Fig. 3E).

No significant difference was observed between blood or lung emTh cells expressing CD11a or P-selectin ligand in all infections, and between CCR5, CCR6 and CXCR3 expressing emTh cells derived from *B. pertussis* and *B. parapertussis* infected mice at either 5 or 25 days p.i. (data not shown).

### *In vitro* migration and adhesion capabilities of blood leukocytes

**Figure 4:**
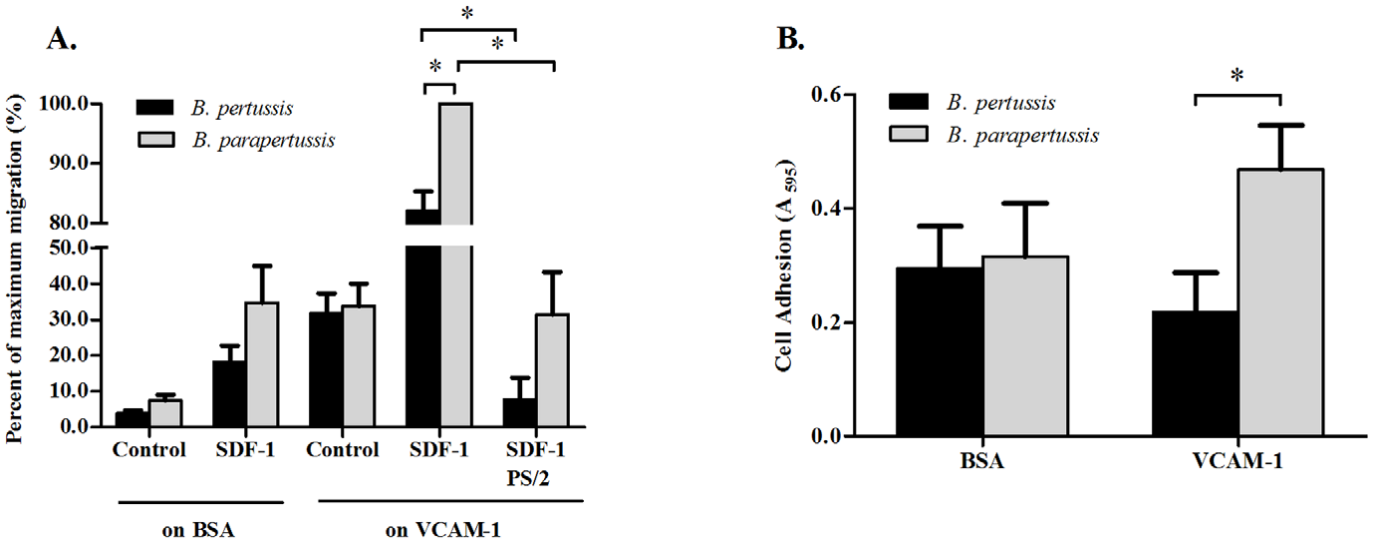
Functional effects of α4 integrins on migration and adhesion of blood leukocytes from *B. pertussis* and *B. parapertussis* infections *in vitro*. (**A**) Migration of blood leukocytes from *B. pertussis* and *B. parapertussis* infections at 5 days p.i. through vascular cell adhesion molecule 1 (VCAM-1)-coated or BSA-coated transwells, in response to stromal cell-derived factor-1 alpha (SDF-1α) or control medium. The number of leukocytes derived from *B. parapertussis* cells through VCAM-1 towards SDF-1α is 100% by definition. Error bars represent the SEM of three independent experiments. In each experiment, four mice were used per treatment group. A two-tailed student's *t*-test was performed; *: p<0.05. (**B**) Adhesion of blood leukocytes at 5 days p.i. to 96-well plates pre-coated with VCAM-1 (10 µg/ml) or BSA, from *B. pertussis* and *B. parapertussis* infections. After 30 minutes, adherents were stained with crystal violet. Values represent mean A_595_ of the solubilized dyes. Error bars represent the SEM of six independent observations. A two-tailed student's *t*-test was performed; *: p<0.05 between *B. pertussis* and *B. parapertussis* infection groups.

To further investigate the differential expression of TRs by circulating emTh cells derived from *B. pertussis* and *B. parapertussis* infection, we assessed the ability of blood lymphocytes to migrate (Fig. 4A) and adhere (Fig. 4B) under *in vitro* conditions. Twenty percent fewer blood lymphocytes derived from mice 5 days p.i. with *B. pertussis* migrated towards SDF-1α through the 3 µm pore transwells coated with VCAM-1 compared to similar blood lymphocytes derived from mice infected with *B. parapertussis* (p<0.05) (Fig. 4A). Pretreatment of cells with PS/2 anti-α4 blocking antibody impeded the migration of lymphocytes derived from both *B. pertussis* and *B. parapertussis* infections, resulting in approximately 70% reduced migration compared to PS/2 untreated cells (p<0.05), further demonstrating the role of α4 integrins (and perhaps CXCR4) in lymphocyte migration to mucosal tissues (Fig. 4A). In addition, adherence of blood leukocytes to VCAM-1-coated surfaces in cells derived from *B. pertussis* infection was 53% less than in cells derived from *B. parapertussis* infection (p<0.05) while no difference was detected in the BSA controls (Fig. 4B).

### Characterization of dendritic cell phenotype

**Figure 5:**
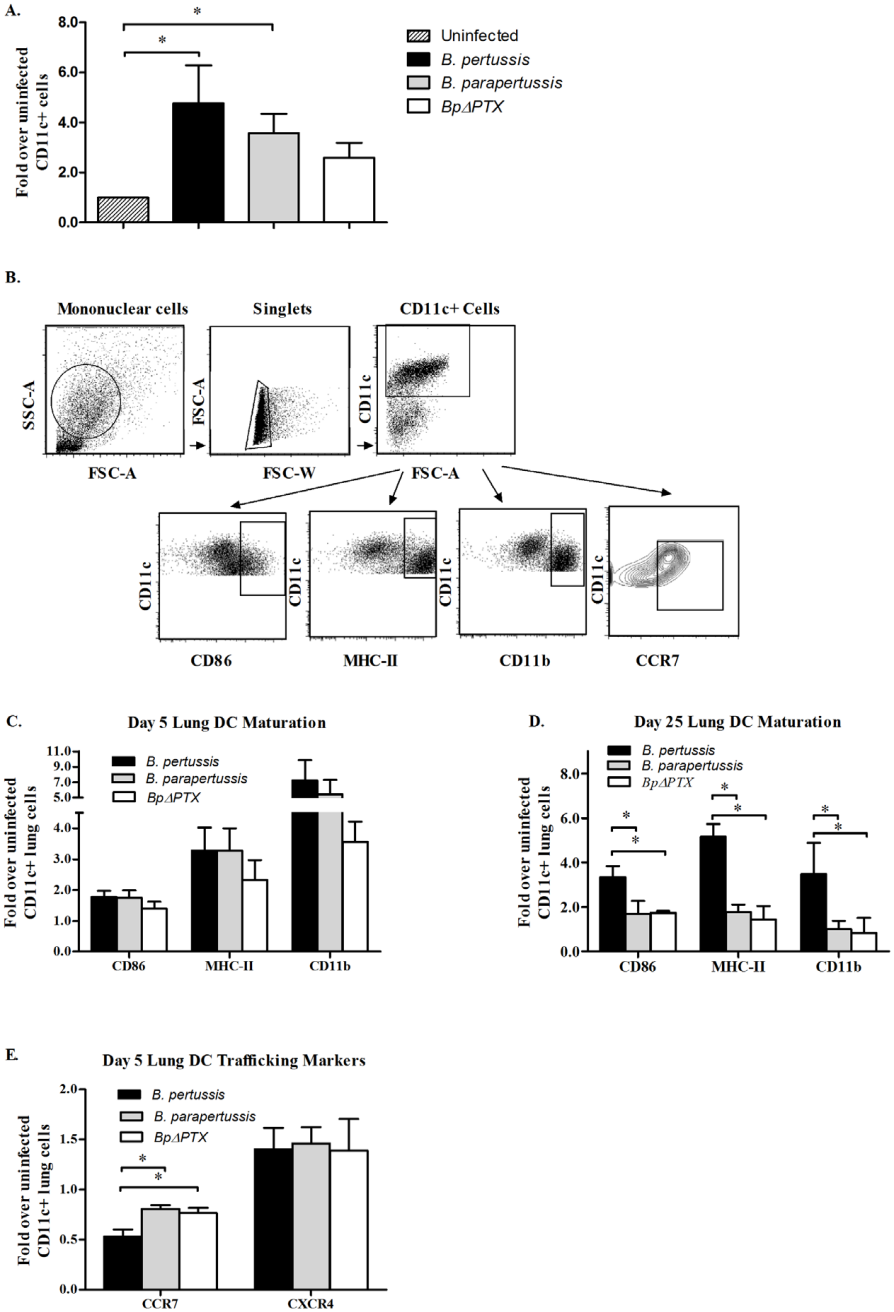
Characterization of lung CD11c^+^ cells during infection with *B. pertussis* and *B. parapertussis*. (**A**) Enumeration of lung CD11c^+^ cells during the early phase of infection with *Bordetella sp*. Mononuclear cells were isolated from lungs and enriched for DCs (CD11c^+^) as detailed. After column enrichment, the total number of CD11c^+^ cells per mouse was estimated using a hemocytometer. (**B**) CD11c^+^ enriched lung mononuclear cells were gated on singlets and then on CD11c^+^ cells (DCs). The frequency of CD11c^+^ cells expressing maturation markers was assessed at 5 days p.i. (**C**) and at 25 days p.i. (**D**), and normalized to the respective cells in uninfected control mice (1.0 by definition). In addition, the frequency of lung CD11c^+^ cells expressing CCR7 and CXCR4 at 5 days p.i. was measured (**E**). The bar charts (**C, D, E**) represent compiled data with error bars showing the SEM of four independent experiments. In each experiment, six mice were used per treatment group. *: p<0.05, and no asterisk indicates p<0.05 as analyzed by a two-tailed student's *t*-test.

Since the imprinting of TRs on T cells is highly dependent on the maturation status as well as the origin and functional characteristics of the DCs [43], [44], we next examined some of these parameters on lung DCs derived from different infection models. An increased number of CD11c^+^ cells were isolated from lungs of mice 5 days p.i. with *B. pertussis* and with *B. parapertussis* compared to uninfected mice (p<0.05). However, the numbers of lung CD11c^+^ cells isolated per mouse did not vary significantly between these two infections (Fig. 5A). While CD11c^+^ cells isolated from *BpΔPTX* infection were increased compared to uninfected controls, this difference did not reach significance (Fig. 5A). Maturation markers were then analyzed on CD11c^+^ cells gated out of lung mononuclear cells as shown in Fig. 5B. The percentage of lung CD11c^+^ cells from *B. pertussis*, *B. parapertussis*, and *BpΔPTX* 5 days p.i. that expressed CD86, MHC class II, and CD11b were all significantly elevated above uninfected cells (p<0.05). However, no significant difference was found between any of the infections (Fig. 5C). In contrast, at 25 days p.i., a high frequency of lung CD11c^+^ cells from *B. pertussis-*infected mice continued to express CD86, MHC class II, and CD11b (4.87-, 3.36- and 3.48-fold over controls respectively) while comparable cells from *B. parapertussis* and *BpΔPTX* infections decreased to corresponding percentages of cells from uninfected mice (Fig. 5D).

We then analyzed whether lung DCs from the infection models differ in their potential capabilities to enter the lung draining lymph nodes, as analyzed by the frequency of CD11c^+^ cells that express CCR7 and CXCR4. Those markers were both previously reported to play a role in this function [45]. A reduced frequency of lung CD11c^+^ cells expressing CCR7 was detected in *B. pertussis*-infected mice compared to cells derived from *B. parapertussis* and *BpΔPTX* infections, which were 0.53, 0.80, and 0.76- fold, respectively, over corresponding cells in uninfected mice (p<0.05) (Fig. 5E). No statistical significance was observed between all infections in the frequency of lung CD11c^+^ cells expressing CXCR4 (Fig. 5E). In addition, no statistically significant difference was observed between the infection groups for CCR7 and CXCR4 at 25 days p.i. (data not shown).

### CCR7 mediated *in vitro* migration of lung CD11c^+^ cells from infected mice

**Figure 6:**
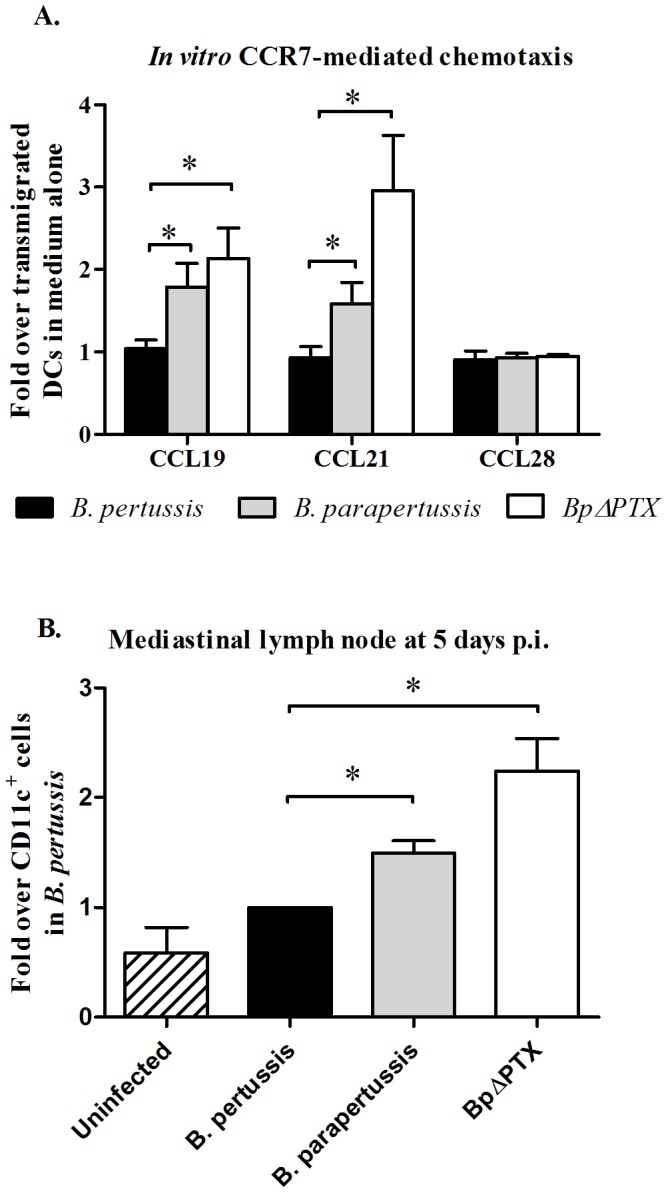
Functional analyses of lung DCs derived from *Bordetella* infections. (**A**) **Transwell CCR7 dependent migration of lung DCs**. 5×10^5^ isolated lung DCs from *Bordetella* infections were placed in the transwell upper chamber and allowed to migrate through a 5 µm polycarbonate membrane in the presence or absence of chemokines. Migrated cells were harvested, stained for CD11c & MHC-II, and enumerated by flow cytometry. Chemotaxis index data are calculated as indicated in Materials and Methods. Depicted are combined data of three experiments. In each experiment, five mice were used per treatment group. *: p<0.05 as analyzed by a two-tailed student's *t*-test. (**B**) **Enumeration of CD11c^+^ cells in the mediastinal lymph nodes.** The mediastinal lymph nodes were harvested, stained with CD11c and CD45, and enumerated with Trubeads by flow cytometry. The absolute number of CD11c^+^ cells from each treatment was normalized to that from *B. pertussis* (1.0 by definition) as detailed in Materials and Methods. The bar chart represents compiled data with error bars showing the SEM of three independent experiments. In each experiment, three mice were used per treatment group. *: p<0.05, and no asterisk indicates p<0.05 as analyzed by the one sample two-tailed student's *t*-test.

To verify that the reduced percentage of lung CD11c^+^ cells expressing CCR7 at 5 days p.i. with *B. pertussis* leads to an inhibited lymphatic migration, we performed an *in vitro* chemotaxis assay towards CCR7 ligands CCL19 and CCL21. At this time point, fewer mature lung DCs (CD11c^+^/MHC-II^+^) from mice infected with *B. pertussis* migrated towards both CCL19 and CCL21 in comparison to those from mice infected with *B. parapertussis* and *BpΔPTX* (p<0.05). This finding suggests that lung DCs from *B. pertussis* infected mice have a compromised CCR7-associated migration. No significant difference was observed in the migration towards CCL28 (ligand to CCR10) between lung DCs derived from the various infections, further emphasizing that these differential observations are CCR7-associated (Fig. 6A).

### Enumeration of CD11c^+^ cells in the mediastinal lymph nodes in *Bordetella* infected mice

To further evaluate if the *in vitro* functional migration analysis described in Fig. 6A bears significance *in vivo*, we enumerated the CD11c^+^ cells present in the lung draining lymph nodes in the various infections. A significant increased number of CD11c^+^ cells were enumerated in the mediastinal lymph nodes harvested from mice infected with *B. parapertussis* and *BpΔPTX* at 5 days p.i., compared to the number of respective cells in *B. pertussis* infection (Fig. 6B), implying a compromised *in vivo* migration of lung DCs from *B. pertussis* infected mice to the draining lymph nodes.

### Trafficking receptor imprinting by lung CD11c^+^ cells derived from *Bordetella* infections

**Figure 7:**
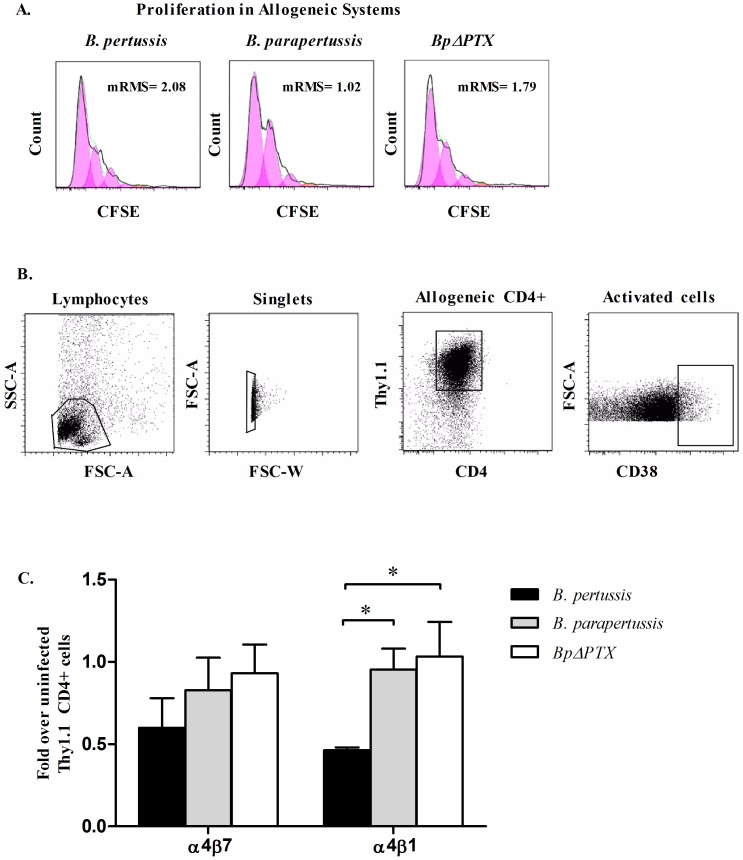
α4β7 and α4β1 imprinting on allogeneic Thy1.1^+^/CD4^+^/CD38^++^ cells. Lung DCs from 5 days p.i. with one of four infection types—*B. pertussis*, *B. parapertussis*, *BpΔPTX*, or uninfected controls—were co-cultured for 4 days with naïve allogeneic Thy1.1^+^/CD4^+^ splenocytes at a 1∶5 ratio. (**A**) Lung DCs from *Bordetella* infections were used to stimulate purified naïve Thy1.1^+^/CD4^+^ splenocytes labeled with CFSE. Histograms depict CFSE dilution profiles. Approximately four cell cycles of division are observed in each system as estimated by FlowJo proliferation analysis. Low mRMS values obtained in every allogeneic system confirmed the accuracy of the analysis. (**B**) Co-cultured cells were gated on lymphocytes, followed by gating on singlets, and then on Thy1.1^+^/CD4^+^/CD38^++^ cells. (**C**) Proportion of Thy1.1^+^/CD4^+^/CD38^++^ cells expressing α4β7 or α4β1 following co-culture with lung DCs derived from *Bordetella* infections was measured and normalized to the respective cells from the uninfected control co-culture (1.0 by definition). The bar charts represent compiled data with error bars showing the SEM of three independent experiments. In each experiment, five mice were used per treatment group. *: p≤0.05, no asterisk: p<0.05.

An allogeneic system was used to assess the ability of lung CD11c^+^ cells derived from all infection groups to imprint mucosal associated TRs on CD4^+^ cells. Lung CD11c^+^ cells, derived from BALB/c Thy1.2^+^ mice at 5 days p.i. with *B. pertussis*, *B. parapertussis* or *BpΔPTX*, were co-cultured with Thy1.1^+^/CD4^+^ splenocytes. The proliferation of allogeneic Thy1.1^+^/CD4^+^ cells was confirmed by a CFSE proliferation assay. Four generations of proliferation were observed in each treatment group over a 4-day period (Fig. 7A). Activated Thy1.1^+^/CD4^+^ cells, as determined by Thy1.1^+^/CD4^+^/CD38^++^ (Fig. 7B), were analyzed for the expression of TRs (Fig. 7C). No significant difference was observed between the percentages of Thy1.1^+^/CD4^+^/CD38^++^ cells out of Thy1.1^+^/CD4^+^ cells in all experimental co-culture systems (data not shown). Compared to the uninfected system, the frequency of α4β7 and α4β1-expressing Thy1.1^+^/CD4^+^/CD38^++^ cells co-cultured with lung DCs from *B. pertussis* infection were 0.6-fold and 0.47-fold over uninfected controls respectively. At the same time, comparable cells co-cultured with lung DCs derived from *B. parapertussis* infection were 0.82-fold and 0.95-fold over, and for *BpΔPTX* were 0.93 and 1.03-fold over uninfected controls, respectively. Only α4β1-expressing Thy1.1^+^/CD4^+^/CD38^++^ cells derived from both *BpΔPTX* and *B. parapertussis* infection differed significantly from *B. pertussis* co-culture cells (p<0.05) (Fig. 7C).

## Discussion

Integrin receptors and other adhesion molecules are often targets for pathogens to enter host cells and to interfere with cellular signaling during bacterial and viral infections [46], [47]. Yet, pathogen interference with the imprinting of integrin receptors on lymphocytes to divert or delay immune cell migration to infected organs is not well documented. In this study, we explored the possibility that interference with TR imprinting on emTh cells may be utilized by *B. pertussis* to prolong survival in the host and delay resolution of the infection.

We compared three murine models of infection with *Bordetella* species: *B. pertussis, B. parapertussis*, and *BpΔPTX*. The first 2 strains are closely related natural human respiratory pathogens that demonstrate 90% genome homology. Still, they markedly differ in the course and duration of respiratory disease and in several important virulence factors. Specifically, *B. parapertussis* does not produce PTX, which is known to interfere with GPCRs and cell migration. To further elucidate the role of PTX, we used the *BpΔPTX* strain for comparison in some of the experiments.

Comparable bacterial loads were initially recovered from both *B. pertussis* and *B. parapertussis* infections. However, *B. parapertussis* had a shorter course of infection that was resolved by day 14 p.i. (Fig. 1), similar to prior observations from Harvill et al [48]. As previously reported by others [11], [15], [49], the initial recovery of *BpΔPTX* from the lungs and recovery at several later time points were about one to two log lower than with *B. pertussis*, emphasizing the role of PTX for efficient colonization by *B. pertussis* as well as the limitations of this model in elucidating PTX-associated immune responses. *B. parapertussis* relies on other bacterial factors for efficient colonization [32]. Since each model of infection has some limitations, we felt that the examination of all three models would provide a more complementary approach to our hypothesis.

To determine temporal fluctuations of the immune response between the different infection models, we examined two time points of infection: early response at 5 days p.i., when high bacterial CFUs were isolated from the lungs (Fig. 1), and late immune response at 25 days p.i., when no bacteria were isolated from the lungs in all infection groups and memory T cell population may rise or predominate. We showed that early in infection with *B. parapertussis* leukocytes were recruited to the lungs (Fig. 2A), and this population of cells was comprised mainly of neutrophils and, to a lesser extent, lymphocytes (Fig. 2B). Furthermore, lung inflammation temporally coincided with an increased frequency of α4β7 and α4β1-expressing emTh cells in the peripheral blood (Fig. 3B), suggesting that an adaptive immune response may have been initiated at this time. Interestingly we did not detect an increase of these cells in the lungs at this time point (Fig. 3D), either because their numbers are low compared to the predominant population of neutrophils or because they have not yet migrated from the peripheral blood to the lung. Similar trends were observed from infection with *BpΔPTX* (Fig. 2A, 2B, 3B, 3D). In contrast, in *B. pertussis* infection, cell infiltration of the lungs at 5 days p.i. was minimal (Fig. 2A, 2B), and the frequency of both peripheral and lung emTh cells expressing α4β7 and α4β1 did not exceed the uninfected control (Fig. 3B, 3D). This suggests that emTh cells may have a limited capacity to enter the infected lungs at this time.

At 25 days p.i. this scenario is reversed. No sign of lung inflammation was observed in *B. parapertussis* infected mice, and the frequencies of both circulating and lung emTh cells expressing α4β7 and α4β1 were similar to those in uninfected controls. Again, infection with *BpΔPTX* closely resembled *B. parapertussis* in regards to the time course of inflammation and the imprinting of TRs, and the recruitment of cells to the lungs. However, at 25 days p.i. with *B. pertussis*, extensive inflammation in the lungs was comprised of T and B cell recruitment (Fig. 2B), corresponding with an increase in both circulating and lung resident emTh cells expressing α4β7 and α4β1. This implies that an effective adaptive immune response is ongoing at this late phase of infection.

These results fall within the wide temporal range of leukocyte recruitment shown to occur in the lungs of mice infected with *B. pertussis* [35], [50]. However, some of our findings contradict other reports that show an increased neutrophil accumulation as early as 48 hours in *B. pertussis* infection, but only a limited neutrophil accumulation in the lungs after infection with a PTX-deficient *B. pertussis* strain [11], [49], [15]. We observed early leukocyte recruitment after *BpΔPTX* infection, and did not observe early neutrophil recruitment after infection with *B. pertussis*. This discrepancy may be due to the 10-fold higher bacterial inoculum used in our study, which perhaps blocked GPCR-associated chemokine migration of neutrophils in *B. pertussis* infection. Additionally, the use of a different *B. pertussis* strain and PTX-deficient derivative may also contribute to the inconsistency between studies.

We further examined functional aspects of altered TR phenotype on emTh lymphocytes. The adhesion molecule, VCAM-1, is expressed on vascular endothelial cells in the lungs [21], and is a common ligand for both α4β1 and α4β7 [51]. Furthermore, SDF-1α is a highly effective lymphocyte chemo-attractant [52] that binds CXCR4, a PTX-sensitive GPCR. The migration of all leukocytes through 3 µm pores of the VCAM-1 coated transwells towards SDF-1α primarily reflects the extravasation and migration of lymphocytes. The results of this assay showed a reduced migration of cells derived from 5 days p.i. with *B. pertussis*-infected mice compared to those derived from *B. parapertussis* infection (Fig. 4A). Although these outcomes may be due to a limited expression of α4β1 and α4β7, other explanations are possible such as a PTX-GPCR inhibition of CXCR4, or a desensitization of CXCR4 associated with PTX-B as previously reported in Jurkat cells [13]. We further showed that lymphocytes from both infections displayed an α4-dependent migration in this system since a dramatic decrease in migration was revealed when α4 was blocked with PS/2 anti-α4. These experiments suggest that lymphocytes derived from early *B. pertussis* infection may be compromised in their α4-dependent extravasation and/or their CXCR4 dependent migration compared to respective cells from *B. parapertussis*.

Since TR imprinting on lymphocytes is dependent on DC maturation and accessibility to secondary lymphoid tissues, we evaluated these characteristics after *Bordetella* infection. In contrast to the report of partial DC maturation and suppressive phenotype during *B. pertussis* infection [53], [54], our study showed that at 5 days p.i. a comparable percentage of lung DCs derived from all infection models up-regulated MHC class II, CD86, and CD11b (Fig. 5C), implying that by 5 days p.i. DC maturation has occurred in all infections and that this process is likely not PTX sensitive. Meanwhile, the expression of these maturation markers persisted at 25 days p.i. only in *B. pertussis* infection, (Fig. 5D) suggesting a prolonged exposure of lung DCs to *B. pertussis* antigens.

The expression of CCR7 (a GPCR) on mature DCs is required for their exit from the peripheral tissues and entrance into the lymphatics [55], [56], [57]. At 5 days p.i., there was a 3 to 4-fold increase in the number of CD11c^+^ cells recruited to infected lungs in *B. pertussis* and *B. parapertussis* infections compared to uninfected lungs (Fig. 5A). However, significantly fewer DCs in the *B. pertussis* infected lungs expressed CCR7 compared to respective cells in *B. parapertussis* and *BpΔPTX* (Fig. 5E). We demonstrated that mature lung DCs from *B. pertussis* infected mice displayed a reduced *in vitro* transwell migration towards CCR7 ligands CCL19 and CCL21 (Fig. 6A). Furthermore, the absolute number of DCs was decreased in mediastinal lymph nodes from *B. pertussis*-infected mice (Fig. 6B) compared to those from *B. parapertussis*- and *BpΔPTX*- infected mice. These results imply that lung DCs in *B. pertussis* infection have a reduced migration capability to the secondary lymph nodes, perhaps due to a PTX inhibitory effect on CCR7-dependent migration. Our results are in agreement with recently reported data by Fedele et al. [58], which show that human monocyte-derived dendritic cells treated with wild type *B. pertussis* exhibit a reduced *in vitro* migration towards CCL21 compared to those treated with *B. pertussis* detoxified strains. Although CXCR4 may also contribute to DC trafficking between tissues and lymphatics [59], no significant difference in the percentage of DCs expressing CXCR4 was observed between our infection groups (Fig. 5E), implying that this GPCR plays a limited role in these respiratory immune responses.

We further showed that lung DCs from *B. pertussis* infection have a reduced capacity to imprint TRs compared to similar cells derived from *B. parapertussis* and *BpΔPTX* infection. This function was assessed in an allogeneic system. Equal proliferation of allogeneic Thy1.1^+^/CD4^+^ cells in all experimental groups was observed in a 4-day co-culture experiment (Fig. 7A), suggesting that efficient interaction between MHC class II and T cell receptors occurred in all of these systems. However, the imprinting of α4β1 TRs on allogeneic T cells varied between the models. A reduced percentage of allogeneic T cells were imprinted with α4β1 following activation with lung DCs derived from *B. pertussis* infection compared to similar models in *B. parapertussis, BpΔPTX*, or uninfected controls (Fig. 7B). This suggests that the nature and origin of DC is responsible for the imprinting of lung associated TRs, as previously shown to occur in the gut [60]. The fact that lung DCs derived from *B. pertussis* infection did not demonstrate a similar imprinting capacity as the other models suggests that PTX-sensitive interactions in the immunological synapse can impair the imprinting of these cells. Although similar trends were observed for α4β7 imprinting, the differences between the allogeneic systems were not statistically significant, suggesting that additional mechanisms may contribute to the alteration of α4β7 expression on emTh cells. For example, it is possible that a leakage of *Bordetella* bacteria/antigens into the esophagus occurred during intranasal infection in addition to the expected delivery of antigens to the trachea. In this circumstance, *Bordetella* antigens may be presented in both gut associated lymphoid tissues by gut-derived DCs that imprint α4β7-expressing emTh cells, as well as in bronchial associated lymphoid tissues by lung-derived DCs that primarily imprint α4β1-expressing emTh cells. This may explain the presence of both α4β7 and α4β1 emTh cell phenotypes in the circulation. Nevertheless, the experimental setting presented in Fig. 7C reflects only lung-associated DC imprinting, and thus imprinting primarily of α4β1-expressing CD4^+^ cells. Thus, the data presented in this study imply that infection with *B. pertussis* inhibits both the migration of lung DCs to secondary lymph nodes and the imprinting of the lung-associated trafficking receptor α4β1 at 5 days p.i.

The data in this study suggest a critical role for emTh cells expressing alpha 4 integrins in the resolution of both *B. pertussis* and *B. parapertussis* respiratory infections. The temporal differential trafficking capabilities of emTh cells in these two infections may be due to an inhibitory effect of PTX. However, our results do not rule out the possibility that other bacterial factors and toxins may contribute to these observations. Future experiments with soluble PTX, such as antigen pulsing experiments, are required to further address whether the imprinting defect is related to the antigen presented or due to a direct effect on the lung DCs ability to effectively interact with CD4^+^ cells in this infection.

In summary, we present evidences that during infection with *B. pertussis* (but not with *B. parapertussis* and *BpΔPTX*) there is a delay in the generation of emTh cells displaying mucosal-associated TRs due to a DC-associated imprinting defect, which may contribute to the prolonged course of whooping cough.
